# Living Environment and Basic Features of the Nematodes Associated with Dung Beetle *Onthophagus atripennis*

**DOI:** 10.2478/jofnem-2025-0035

**Published:** 2025-08-31

**Authors:** Yuya Ikeda, Natsumi Kanzaki, Ryoji Shinya

**Affiliations:** School of Agriculture, Meiji University, Kawasaki, Kanagawa, Japan.; Kansai Research Center, Forestry and Forest Products Research Institute, Fushimi, Kyoto, Japan

**Keywords:** insect-nematode interaction, dung beetle, ecology, viviparity

## Abstract

Viviparity is a very rare reproductive mode in nematodes, having been documented in only six species. Five of these species have been isolated among the dung beetles *Onthophagus*, suggesting that studying the environments associated with dung beetles may help shed light on why viviparity evolved in these particular species. *Onthophagus atripennis* is often closely associated with the viviparous nematode *Tokorhabditis atripennis*, as well as some other oviparous nematodes. Hence, the system involving *T. atripennis* and dung beetles could provide valuable insights into the adaptive significance of viviparity in nematodes. To explore this idea, it is essential to first gain a better understanding of the natural habitats of *T. atripennis*, which remain poorly understood. Therefore, we investigated the nematode communities associated with *O. atripennis* and identified potential habitats for *T. atripennis* in environments used by dung beetles. Nematodes associated with *O. atripennis* and those inhabiting its rearing cases were isolated from samples. Two *Tokorhabditis* species were isolated from the rearing cases of *O. atripennis*, suggesting that they inhabit environments used by *O. atripennis*. Regarding other oviparous nematodes, some appeared to have relatively strong associations with dung beetles, but more detailed studies are needed to confirm their specific habitats. Although further investigations are necessary, the fact that nematodes, including viviparous species, were isolated from environments used by dung beetles provides important information about the potential competitors or predators of *T. atripennis* in such environments.

Obligate viviparity is a reproductive mode in which embryonic development occurs within the reproductive system (ovary or sexual duct), body cavity (coelom, pseudocoel, or hemocoel), parental tissues, or tissue-like layers with nutrient supply (parenchyma, mesohyl, or mesoglea), resulting in live births (Ostrovsky et al., 2016). This reproductive mode is distinguished from facultative viviparity (in which oviparous species retain their offspring in their utero still they hatched to reflect their environments) and ovoviviparity (in which young are born without nutrient supply after hatching in utero) (Yamashita et al., 2023). This reproductive strategy is very rare in nematodes; only two genera (six species in total) are believed to have obligate viviparity (Herrmann et al., 2013; Kanzaki et al., 2017, 2021; Ragsdale et al., 2022). Within these six species, five of them have been found in association with *Onthophagus* dung beetles (Herrmann et al., 2013; Kanzaki et al., 2017, 2021; Ragsdale et al., 2022).

This intriguing pattern suggests that nematode viviparity may have evolved as an adaptation to the unique ecological conditions created by dung beetles, along with their chemical and physical habitats and cohabitants. For example, *Onthophagus* dung beetles create brood balls, which are sealed chambers made from animal feces that provide a food source for their larvae, and viviparous nematodes may have adapted to also use these enclosed environments. The specific ecological and physiological advantages that viviparity confers to these nematodes remain unclear. In particular, fundamental information necessary for establishing dung beetle-nematode study systems — such as the life cycle of viviparous nematodes under natural conditions and potential co-habiting species that may affect their population dynamics (e.g., competitors or predators) — is still lacking.

*Onthophagus atripennis* hosts oviparous and viviparous nematodes, is widely distributed, and can be easily sampled across Japan. Ikeda et al. (2024) demonstrated that *Tokorhabditis atripennis* is widely distributed in Japan and is associated with the dorsum of the wings and the mesothorax of *O. atripennis*. This suggests that it is closely associated with the beetle, relying on it for transport to new habitats. These findings highlight the potential of the *T. atripennis*-*O. atripennis* system for investigating the adaptive significance of viviparity in nematodes.

To explore the ecological significance and evolutionary origins of viviparity, it is necessary to understand the natural habitat of *T. atripennis*, including its environmental conditions, competitors, predators, and symbionts. Because few studies have reared *O. atripennis* under laboratory conditions (Kishi & Nishida, 2006; Kishi, 2014), it is crucial to verify whether this beetle can reliably be used in nematode experiments before establishing a controlled experimental system.

Therefore, in this study, we aimed to (1) characterize the natural environment of *T. atripennis*; (2) identify the nematode fauna associated with dung beetle environments; and (3) explore the potential of *O. atripennis* rearing cases as a basis for future laboratory-based research on dung beetle-nematode associations. We first isolated and attempted to culture nematodes from beetles to identify the oviparous nematode fauna associated with *O. atripennis*. Then, to determine the habitat preferences of these nematodes, we reared *O. atripennis* under laboratory conditions, and collected feces, brood balls, and soil from the rearing cases. We then isolated and cultured nematodes from these samples. From this we obtained the nematodes *Onthodiplogaster japonica* — isolated from *O. atripennis* — along with two viviparous species and four oviparous species isolated from cow dung and brood balls in the rearing cases of *O. atripennis*. Additionally, we successfully obtained six brood balls from two of the four rearing cases.

## Materials and Methods

### Isolation of nematodes from dung beetles

Our geographical survey of *T. atripennis* also isolated oviparous nematodes associated with *O. atripennis* in the Kanagawa prefecture and described the procedure involved (Ikeda et al., 2024). Here, we describe this oviparous nematode’s data. Briefly, beetles were caught using pit-hole traps using rotten fishmeal as an attractant. The traps were established at two separate locations in Kawasaki, Kanagawa Prefecture at the Ikuta Campus of Meiji University (35°36′39.8″N, 139°32′55.8″E; May 14, August 2–4, and September 16, 2021) and at the Meiji University Kurokawa Field Science Center (GPS: 35°36′31.5″N, 139°27′20.8″E; September 30 and October 20–21, 2021). The beetles were dissected on a Syracuse watch glass, and any nematodes found were transferred onto nematode growth medium (NGM) (Brenner, 1974), or NGM with two small pieces of dog food medium (dog food medium or DFM: 20 g crushed dog food, 4 g agar, and ion-exchange water). These samples were seeded with *Escherichia coli* OP50 and incubated at 25°C (Ogura and Mamiya, 1989).

### Beetle rearing and nematode isolation from rearing cases

**Figure 1:**
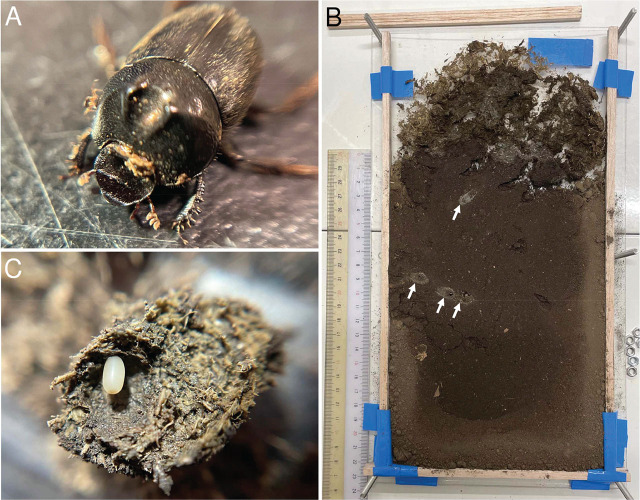
*Onthophagus atripennis* and their brood balls. (A) An adult male of *Onthophagus atripennis*. (B) A rearing case of *Onthophagus atripennis* and their brood balls (white arrow). (C) A brood ball and egg of *Onthophagus atripennis*.

To elucidate the habitat preferences of dung beetle-associated nematodes, 14 adult *O. atripennis* were separated into four arbitrary groups and transferred to rearing cases constructed in our laboratory (Figs. 1 and S1; Table 2). We constructed these rearing cases with an 8-mm-thickness wooden frame between 2 acrylic boards (320 × 180 × 2 mm), fixed by a bolt and nut to part of the acrylic boards’ edge (Fig. 1, Fig. S1). Prior to use, the cases were sterilized with boiled water and filled with autoclaved chernozem soil, adding water sufficient for a mass rate of around 20% until the mixture reached approximately a 200-mm depth. Subsequently, 50 g cow feces (frozen at −25ºC to kill nematodes and defrosted before use) were added on the top of the soil (Fig. S1). Three to four dung beetles (*O. atripennis*) were transferred to each case (Table 2). Those dung beetles had been caught using pit-hole traps set in Ikuta Campus of Meiji University, Kawasaki, Japan, and moved to the cases in 48 hours after collection.

**Table 1:** *Onthopahgus atripennis* collected by pit-hole trap in this study and the associated nematodes. All beetles were collected in Kawasaki, Kanagawa.

**Date**	**Site**	**Associated nematode**	**Strain**
2021.5.14	Meiji University	-	-
2021.5.14	Meiji University	*Tokorhabditis atripennis*	SHR9
2021.5.14	Meiji University	-	-
2021.5.14	Meiji University	-	-
2021.5.14	Meiji University	-	-
2021.5.15	Meiji University	-	-
2021.5.15	Meiji University	-	-
2021.8.2	Meiji University	-	-
2021.8.2	Meiji University	-	-
2021.8.2	Meiji University	-	-
2021.8.2	Meiji University	*Onthodiplogaster japonica*	SHR18
2021.8.4	Meiji University	*Tokorhabditis atripennis*	SHR21
2021.8.4	Meiji University	-	-
2021.9.30	Kurokawa Field Science Center	*Onthodiplogaster japonica*	SHR154
2021.9.30	Kurokawa Field Science Center	*Tokorhabditis atripennis*	SHR16
2021.9.30	Kurokawa Field Science Center	-	-
2021.9.30	Kurokawa Field Science Center	-	-
2021.9.30	Kurokawa Field Science Center	-	-
2021.9.30	Kurokawa Field Science Center	-	-
2021.9.30	Kurokawa Field Science Center	-	-
2021.9.30	Kurokawa Field Science Center	*Onthodiplogaster japonica*	SHR22

**Table 2:** The details of beetles’ rearing cases and samples isolated from the cases.

**ID**	**Date of setting**	**Date of dissection**	**Number of beetles**		**Number of Brood balls**

			**Male**	**Female**	
1	2023.7.14	2023.7.21	2	2	2
2	2023.7.14	2023.7.21	1	2	4
3	2023.7.16	2023.7.24	1	2	0
4	2023.7.18	2023.7.25	2	2	0

After one week, we opened the rearing cases and collected cow dung, soil around beetle tunnels, beetle brood balls, and any soil in the cases that was not adjacent to the dung, tunnels, or brood balls. We confirmed that all collected brood balls contained either an egg or a larva. To isolate nematodes from these samples, each sample was placed on NGM + DFM seeded with *E. coli* OP50 and incubated at 25 °C. When adults were observed in the medium, each nematode was transferred onto new NGM + DFM seeded with *E. coli* OP50 and incubated again at 25ºC. After the transferred nematodes’ propagation, we identified the nematodes based on their morphological characters and molecular profiles.

### Light microscopy

Adult nematodes from each strain were washed in deionized water or M9 buffer (3 g KH_2_PO_4_, 9 g Na_2_HPO_4_, 5 g NaCL, and H_2_O to a volume of 1 L, in an autoclaved vessel), and mounted on a 2% agar pad with a drop of liquid, covered with a cover slip, and killed via exposure to heat. Then, the nematodes were observed under a light microscope equipped with differential interference contrast optics (Eclipse 80*i*, Nikon, Tokyo, Japan).

### Molecular profiles and phylogeny

To establish the molecular profiles of the strains obtained in this study, ribosomal DNA (rDNA) segments were sequenced (Table 3). Additionally, partial 18S and 28S rDNA segments were used to confirm species status. The DNA of each strain was prepared using Direct PCR Lysis Reagent (Viagen Biotech, Los Angeles, CA, USA) or ISOHAIR (Nippongene, Tokyo, Japan) (Kikuchi et al., 2009; Tanaka et al., 2012). The rDNA segments were amplified and sequenced using the universal primers shown in Table 3 (DeLey et al., 1999; Holterman et al., 2006; Carta and Li, 2018; Kanzaki et al., 2021). The obtained DNA fragments were purified using either ExoSAP-IT PCR Product Cleanup Reagent (Thermo Fisher Scientific, Waltham, MA, USA) or Sephacryl S-300 HR spin column (Cytiva, Marlborough, MA, USA), following the manufacturers’ respective instructions. Samples were either sequenced according to the method of Ekino et al. (2017) or submitted to Macrogen Japan Corp. (Tokyo, Japan) for sequencing from both strands using the same polymerase chain reaction (PCR) primers. The sequences obtained were confirmed and edited manually using ApE v3.1.6. Finally, we identified the strains through Basic Local Alignment Search Tool (BLAST) homology searches. The obtained sequences were deposited in the GenBank database under the accession numbers PV022049-PV022058 and PV919073.

**Table 3:** The nematode strains isolated from samples of rearing cases. The ID number described in “Isolated from” is the “ID” used in Table 2.

**Strain**	**Isolated from**	**Estimated taxon**	**Sequence region (Primer)**	**Accession No.**
SHR88	Feces from ID 1	*T. atripennis*	28S rDNA (D1F/D4R), 18S	PV022059
			rDNA (18S-CL-F3/18S-CL-R1)	PV022047
SHR142	Tunnel from ID 1	*T. atripennis*	28S rDNA (D1F/D4R), 18S	PV022060
			rDNA (18S-CL-F3/18S-CL-R1)	PV022048
SHR133	Feces from ID 2	*Pelodera* sp.	28S rDNA (D2a/D4R), 18S	PV022061
			rDNA (988F/F22/R13/2646R)	PV022049
SHR132	Feces from ID2	*T. atripennis*	28S rDNA (D1F/D4R), 18S	PV022062
			rDNA (18S-CL-F3/18S-CL-R1)	PV022050
SHR93	Tunnel from ID2	*T. atripennis*	28S rDNA (D1F/D4R)	PV022051
SHR120	Tunnel from ID 2	*Oscheius* sp.	18S rDNA (18S-CL-F3/18S-CL-R1)	PV022063
SHR108	Brood ball from ID 2	*T. atripennis*	28S rDNA (D1F/D4R), 18S	PV022064
			rDNA (18S-CL-F3/18S-CL-R1)	PV022052
SHR107	Tunnel from ID 3	*T. tauri*	28S rDNA (D1F/D4R), 18S	PV022065
			rDNA (18S-CL-F3/18S-CL-R1)	PV022053
SHR143	Feces from ID 3	*Mononchoides* sp.	28S rDNA (D1F/D2a/D3b/D4R), 18S	PV022066
			rDNA (18S-CL-F3/18S-CL-R1)	PV022054
SHR106	Feces from ID 4	*T. atripennis*	28S rDNA (D1F/D4R), 18S	PV022067
			rDNA (18S-CL-F3/18S-CL-R1)	PV022055
SHR150	Feces from ID 4	*Pelodera* sp.	28S rDNA (D1F/D4R), 18S	PV022068
			rDNA (18S-CL-F3/18S-CL-R1)	PV022056
SHR114	Tunnel from ID 4	*T. atripennis*	28S rDNA (D1F/D4R), 18S	PV022069
			rDNA (18S-CL-F3/18S-CL-R1)	PV022057

To confirm our molecular identifications, we conducted maximum likelihood (ML) phylogenetic analyses based on 28S rDNA. Multiple sequence alignments were conducted using MAFFT v7.490 (Katoh and Standley, 2013) with the ‘--auto’ option. The aligned sequences were trimmed using ClipKit v1.4.1 (Steenwyk et al., 2020) in ‘kpic-smart-gap’ mode. We then deleted regions lacking sequences using Jalview v2.11.4.0 (Waterhouse et al., 2009) and used these edited sequences for phylogenetic analyses. MEGA v11.0.13 (Tamura et al., 2021) was used to construct and analyze phylogenetic trees.

### Categorizing the reproductive modes of nematodes

After the identification of each species, we categorized its reproductive mode based on both our observation and previous descriptions. When nematodes laid eggs on culture media, they were categorized as “oviparous,” but if the nematodes did not lay any eggs and kept juveniles in their uterus, these were considered either viviparous or ovoviviparous (the reproductive mode in which juveniles hatch in their mother’s utero but do not receive any nutrition from their mother after hatching). These two modes could be distinguished based on the observation of embryonic development, as well as previous descriptions.

## Results

### Isolation of oviparous nematodes from *O. atripennis*

**Figure 2:**
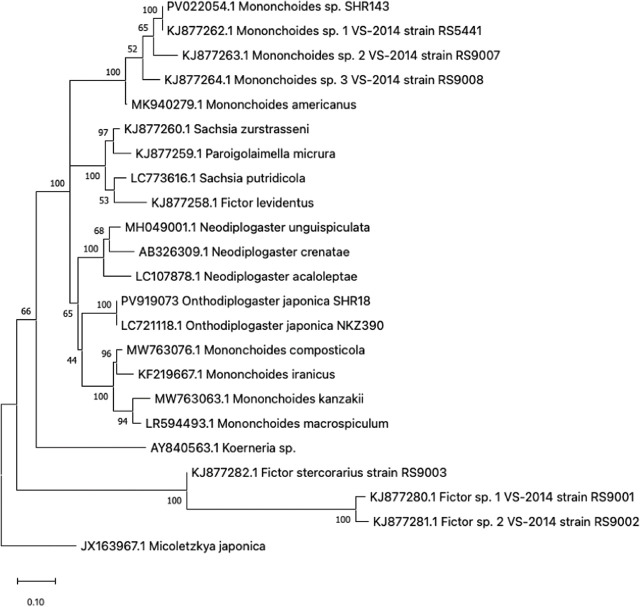
Phylogenetic tree of *Mononchoides* sp. (SHR143) and *Onthodiplogaster japonica* (SHR18) with 20 Diplogastrid taxa inferred from the 28S rDNA gene by using the Maximum Likelihood method under Tamura-Nei model. Numbers at the nodes represent support values in percent for 1000 bootstrap replicates.

In all, 21 *O. atripennis* were collected from pit-hole traps, and oviparous nematodes were isolated from three individuals (Table 1). All were recovered from the mesothorax of the beetle. The partial 28S rDNA sequences of three strains (SHR18, 27, and 154) isolated from these individuals were determined and found to be identical in their overlapping region (Fig. S2). Phylogenetic analyses suggest that SHR18 is conspecific to *O. japonica* NKZ390 (Kanzaki et al., 2023) (Fig. 2). Accordingly, these three strains were identified as *O. japonica*. The typological characters of these strains also match those of *O. japonica*.

### Isolation of nematodes from rearing cases

We found and collected six brood balls from two rearing cases. Twelve nematode strains were obtained from nine sample locations, including cow dung, soil around tunnels, and brood balls (Table 3) — however, no nematodes could be isolated from soil from within cases. Based on 18S and 28S rDNA sequences, we identified two species of viviparous nematode and four species of oviparous nematode (Table 3). *Onthodiplogaster japonica* was not identified in this experiment. Except for SHR107, all strains were successfully cultured on NGM seeded with *E. coli* OP50.

### Two species of viviparous nematode from genus *Tokorhabditis*

**Figure 3:**
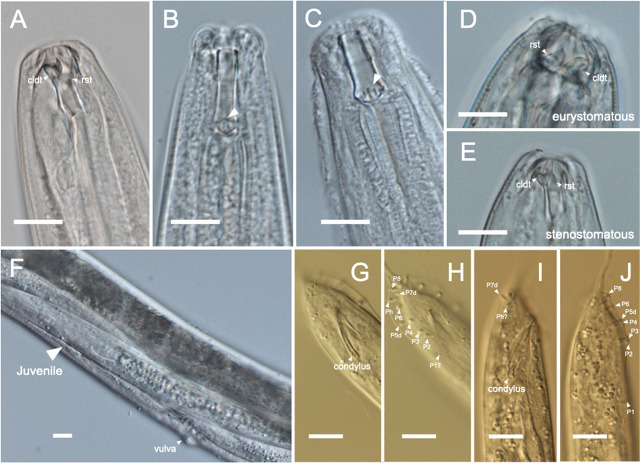
Characteristic parts of nematode isolated in this study. Head regions of (A) *Onthodiplogaster japonica* (SHR18), (B) *Pelodera* sp. (SHR133), (C) *Pelodera* sp. (SHR150), and (D) eurystomatous form and (E) stenostomatous form of *Mononchoides* sp. (SHR143). (F) Ventral part and a juvenal of *Tokorhabditis atripennis* (SHR108). Mail tail regions of (G, H) *Tokorhabditis atripennis* (SHR108), and (I, J) *Tokorhabditis tauri* (SHR107). The white bars in these images are scale of 10 μm. cldt: claw-like dorsal tooth. rst: right subventral tooth.

**Figure 4:**
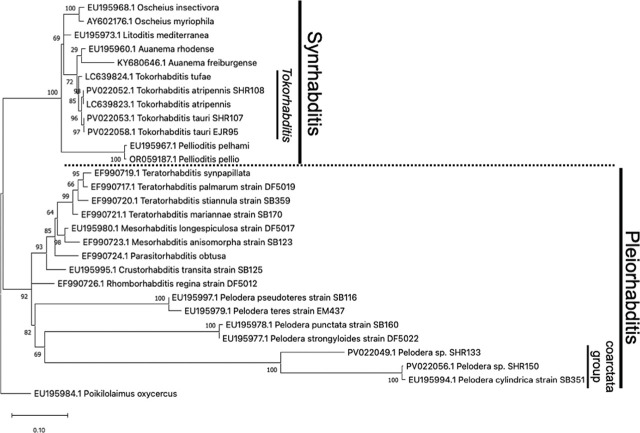
Phylogenetic tree of *Tokorhabditis* spp. with 7 syn. Rhabditis taxa and *Pelodera* spp. with 9 Pleiorhabditis taxa inferred from the 28S rDNA gene by using the maximum likelihood method under GTR + G + I model. Numbers at the nodes represent support values in percent for 1,000 bootstrap replicates.

Based on the sequences of nematode strains, we identified *T. atripennis* from three cow dung samples (SHR88, 132, and 106), three soil samples around tunnels (SHR142, 93, and 114), and one brood ball (SHR108). We also identified another viviparous species, *Tokorhabditis tauri* (SHR107), from one soil sample around a tunnel. However, the latter species had only previously been reported in North America (North Carolina, Indiana, and Florida) (Ragsdale et al., 2022). Therefore, to confirm the species, we observed the morphological features of our specimen, compared its rDNA sequences to those of US strains of *T. tauri* (EJR13, accession numbers LC639822.1 and EJR95), and constructed a phylogenetic tree based on 28S rDNA sequences. All of these investigations confirmed our identification of *T. tauri* (Figs. 3, 4, and S3). In those nematodes’ cultures, developing embryos were observed in adults’ uteri, and no eggs were served; thus, according to the species’ description in a previous study (Ragsdale et al., 2022), we concluded they were obligate viviparous nematodes (not ovoviviparous).

### An omnivorous nematode, *Mononchoides* sp., isolated from cow dung

An omnivorous nematode (SHR143) was isolated from cow dung. Based on its rDNA sequence and its phylogenetic status within diplogastrid nematodes, it appeared conspecific to *Mononchoides* sp. RS5441, which was isolated from a *Geotrupes* dung beetle in France (Fig. 2) (Mayer et al., 2009; Susoy et al., 2015). The strain was found to belong to the group that includes *Mononchoides americanus*. Its morphological features were in line with *Mononchoides* (Figs. 3 and S4; Fürst von Lieven and Sudhaus, 2002; Calaway and Tarjan, 1973; Fürst von Lieven and Sudhaus, 2003). We observed eggs in this nematode’s culture, so we concluded this nematode SHR143 was an oviparous species.

### Other bacteriovorous and oviparous nematodes

**Figure 5:**
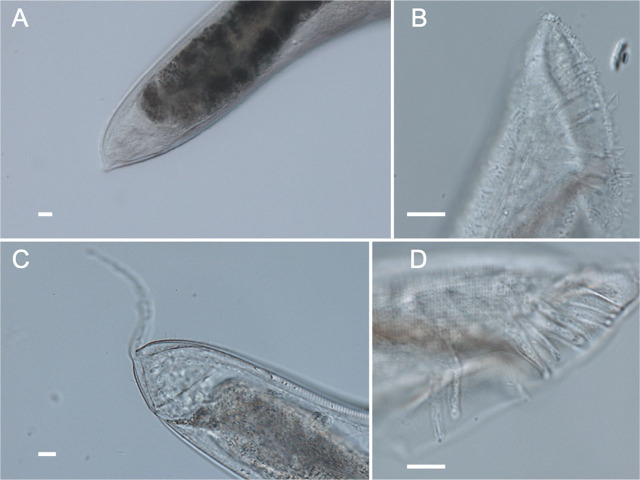
Tail regions of Pelodera (coarctata group) isolated in this study. (A) The female tail of SHR133. (B) The male tail of SHR133. (C) The female tail of SHR150. (D) The male tail of SHR150. The white bars in these images are scale of 10 μm.

Bacteriovorous, oviparous nematodes were isolated from cow dung (SHR133, 150) and soil around tunnels (SHR120). These were genetically identified as *Oscheius* sp. (SHR120) and two species of *Pelodera* belonging to the *coarctata* group (SHR133 and 150) (Figs. 3–5). Morphologically, they also fit the *Pelodera* genus (Figs. 3 and 5) (Sudhaus, 2011). Many eggs were observed in those nematodes’ cultures, leading us to conclude that they were oviparous nematodes.

## Discussion

We isolated *O. japonica* from three *O. atripennis* individuals, along with two viviparous and four oviparous nematode species from rearing cases. These results provide new insights into the diversity of nematodes associated with *O. atripennis* and their potential ecological interactions.

In a previous study, *O. japonica* was also isolated from *Onthophagus* in Kyoto, Japan, but the location on the beetle’s body was not specified (Kanzaki et al., 2023). Therefore, our isolation of *O. japonica* from the mesothorax beneath the elytra of three *O. atripennis* is the first report of its localization on the beetle body; it is also the first report from Kanagawa. Considering that the species has been found at several localities in Yamagata and Ibaraki Prefectures (Kanzaki, unpublished observation), *O. japonica* is likely to be widely distributed in the carrier beetle’s habitat; due to its wide feeding habitat, it could possibly affect *T. atripennis* as competitor and/or predator (Kanzaki et al., 2023). Nevertheless, we did not isolate *O. japonica* from rearing cases in the present study, and we do not have a clear explanation for this result. It is possible that the experimental period (one week) may have been too short to detect *O. japonica*, or that our sample may have been too small; nematodes were not found on every single beetle, and we examined only 14 individuals. Further detailed studies employing various conditions and larger sample sizes are necessary to clarify the biological interactions of this nematode species.

Two viviparous nematode species, *T. atripennis* and *T. tauri*, were isolated from rearing-case samples. The former was found in three sample types (cow dung, soil samples around tunnels, and brood balls), suggesting that it reproduces where *Onthophagus* beetles feed and breed, colonizing such environments via their association with these beetles. The other species was found from a soil sample collected from a beetle tunnel. This is the first recorded isolation of *T. tauri* outside of North America, and it suggests that the distribution range of the species is wider than previously reported. Both of these species have previously been isolated from *Onthophagus* beetles, and Ragsdale et al. (2022) mentioned a high density of *T. tauri*. Therefore, we speculate that these two *Tokorhabditis* species regularly colonize environments inhabited by *Onthophagus* beetles.

We successfully cultured four nematode species on NGM plates seeded with *E. coli*. *Mononchoides* sp. shows stomatal dimorphism, and the predatory form appeared in our cultures, suggesting that the species is an omnivore that is able to feed on both bacteria and nematodes. Therefore, it may be both a competitor to, and a predator of, *Tokorhabditis* and other bacteriophagous nematodes. However, few associations between *Mononchoides* and dung beetles have been reported.

*Mononchoides ahpodii* has been reported as a symbiont of *Aphodius* dung beetles (Poinar et al., 1976; Sudhaus and Fürst von Lieven, 2003; Mahboob and Tahseen, 2021), and an undescribed species, *Mononchoides* sp. RS5441, has been isolated from *Geotrupes* sp. (Mayer et al., 2009; Susoy et al., 2015; also see isolation information deposited in GenBank). To our knowledge, *Mononchoides* has never been isolated from *Onthopahgus* beetles (Kanzaki et al., 2023; Ikeda et al., 2024). *Mononchoides* nematodes have often been isolated from fecal samples (Sudhaus and Fürst von Lieven, 2003); therefore, it is possible that the *Mononchoides* sp. isolated in this study had a facultative association with *O. atripennis* and was primarily carried by other invertebrates collecting the dung.

Additionally, our conclusion that *Mononchoides* sp. SHR143 is conspecific to strain RS5441 suggests that SHR143 may have originated from *Phelotrupes* dung beetles. This strain was originally isolated from a *Geotrupes* dung beetle (Mayer et al., 2009; Susoy et al., 2015), but *Geotrupes* beetles are not distributed in Japan. If *O. atripennis* is not the primary host of *Mononchoides* sp. SHR143, SHR143 may be associated with other relatives of *Geotrupes* beetles. In Japan, *Phelotrupes*, a genus related to *Geotrupes*, is frequently found cohabiting with *Onthophagus* dung beetles. Therefore, it is possible that the *Mononchoides* sp. is primarily associated with *Phelotrupes* in Japan and facultatively associated with *O. atripennis*. *Mononchoides* has not yet been isolated from *Phelotrupes* dung beetles, however, so further nematode surveys focusing on larger samples of dung beetle species will be necessary to determine their natural carrier associations.

Two *Pelodera* species were isolated from rearing cases. Although the rDNA sequences were not identical to any deposited in the National Center for Biotechnology Information database, based on a BLAST search, molecular phylogenetic analyses, morphological features, and the habitats from which they were collected, we concluded that they belong to the *coarctata* group. The *coarctata* group consists of 14 species, 11 of which have been isolated from dung or dung beetle genera, including *Aphodius*, *Geotrupes*, *Catharsius*, *Onthophagus*, and *Digitonthophagus* (Leuckart, 1891; Völk, 1950; Sachs, 1950; Sudhaus, 2011; Mahboob et al., 2023; Sudhaus, 2023). Sachs (1950) isolated five species of *Pelodera* nematodes from cow dung, along with 18 dung beetle species, two Hydrophilidae beetle species, four rove beetle species (Staphilinidae), and three Histeridae beetle species. Additionally, Weller et al. (2010) reported that more than 50% of *Geotrupes stercorosus* were associated with *Pelodera* species. However, we did not directly isolate *coarctata* group species from *O. atripennis*. In Japan, *Phelotrupes* are often collected with *O. atripennis* in pit-fall traps or from natural habitats (e.g., animal feces), such that the *Pelodera* species obtained in our study may be derived from *Phelotrupes* but also associated with *O. atripennis*.

The *Oscheius* sp. obtained in this study was closely related to *Oscheius tipulae*, based on its 18S rDNA sequence (99.22% identity). This nematode likely originates from soil and was probably facultatively associated with *O. atripennis*, with which it does not seem to have a robust relationship. Anderson and Sudhaus (1985) described *O. dolichuroides* as being associated with the larva of a scarab beetle collected from decaying matter in a tree hole, indicating that this larva was not that of a dung beetle. Other *Oscheius* species have frequently been isolated from soil and soil-inhabiting arthropods, but have never been found in association with dung beetles (Lam and Webster, 1971; Andrássy, 1984; Anderson and Sudhaus, 1985; Poinar, 1986; Smart and Nguyen, 1994; Sudhaus and Hooper, 1994; Stock and al., 2005; Tahseen and Nisa, 2006; Kito and Ohyama, 2008; Khanum and Fayyaz, 2008; Zhang et al., 2008; Gorgadze, 2010; Lisnawita et al., 2010; Sudhaus, 2011, 2023). However, Sudhaus and Hooper (1994) showed that *Oscheius guentheri* thrives in cow dung, and that its third-stage juveniles actively live on the dung surface. Based on these observations, they concluded that this nematode is dispersed phoretically by insects inhabiting the same environment, which suggests that *Oscheius* species can thrive in dung environments and associate with dung beetles. Future research may further elucidate the relationships between *Oscheius* and dung beetles.

In this study, all isolated nematodes were cultured using nematode growth medium (NGM) or NGM with two small pieces of dog food medium. Although these media are useful for culturing bacterivorous and omnivorous nematodes, it is possible that some species — such as fungivorous nematodes or bacterivorous species that do not prefer *E. coli* OP50 — cannot be successfully cultured this way. To elucidate unculturable species or those that require special conditions for propagation, more detailed and comprehensive methods, such as amplicon sequence analysis, are needed in future studies.

We successfully obtained six brood balls from two rearing cases and isolated several nematode species, which suggests that *O. atripennis* can be propagated and that nematodes within these cases can be tracked in a laboratory setting. Although further investigation is needed to confirm these suggestions, this rearing-case system is expected to be useful for verifying the structure of nematode fauna in dung environments and the adaptive advantages of viviparity in nematodes.

*Tokorhabditis* and *Sudhausia* are the other viviparous genera have been isolated from environments used by *Onthophagus* beetles (Herrmann et al., 2013; Kanzaki et al., 2017, 2021; Ragsdale et al., 2022), which suggests that nematode vivipary is driven by the abiotic and biotic factors of dung environments. These may include high concentrations of nitrogen and phosphate compounds, or biological interactions surrounding the beetles between bacteria, fungi (yeasts), mites, nematodes, and other microbes and meiofauna. Many rhabditid nematodes have facultative viviparity (endotokia matricida, bagging), the reproductive mode in which oviparous species retain mature eggs and give live birth to young that hatch from rigid eggs in utero (Mitchell et al., 1979; Johnigk and Ehlers, 1999a; Johnigk and Ehlers, 1999b). This is facilitated by aging of the parents, some abiotic factors, and biotic factors, such as the presence of bacteria harmful to nematodes, starvation, and osmotic pressure (Chen and Caswell-Chen, 2004; Mosser et al., 2011). Continuous exposure to such factors in dung environments may facilitate the evolution of facultative viviparous nematodes to obligate viviparity. In this study, an experimental system (rearing cases) was established, and related nematodes were isolated as cultures. All the nematodes in this study were recognized as belonging to Diplogastridae or Rhabditidae (Synrhabiditis group and *Pelodera coarctata* group), and this result was basically consistent with the previous studies that investigated dung beetles’ nematodes (Bovien, 1937; Sachs, 1950; Weller et al., 2010). Interestingly, some *Pelodera* spp. are considered to have facultative viviparity, which is in accordance with the hypothesis that dung beetle environment drives vivipary (Mahboob, 2023).

However, so far, information concerning dung beetle-associated nematode fauna is limited, and many undescribed species still exist. In addition, the sample size in this study was not enough to determine the dung beetle’s nematode fauna. Further comprehensive approaches are necessary to examine the nematode fauna and ecological origins of viviparity. Nematode interactions could be investigated through co-culture (competition) studies using the materials obtained in this study. Microbes and meiofauna should additionally be examined for their potential roles in competition with and predation of viviparous nematodes. Molecular analyses, including amplicon sequencing, along with further intensive isolations followed by culturing and molecular identification, may enable more detailed examinations.
